# Thyroid disorders and the incidence of type 2 diabetes: insights from a 10-year cohort study in Germany

**DOI:** 10.1530/EC-24-0554

**Published:** 2025-01-31

**Authors:** Theresia Sarabhai, Karel Kostev

**Affiliations:** ^1^ Department of Endocrinology and Diabetology, Medical Faculty and University Hospital Düsseldorf, Heinrich-Heine-University, Düsseldorf, Germany; ^2^ Epidemiology, IQVIA, Frankfurt am Main, Germany

**Keywords:** thyroid, hypothyroidism, hyperthyroidism, diabetes

## Abstract

**Background:**

Thyroid dysfunctions, such as hypothyroidism and hyperthyroidism, are known to influence metabolism, but their long-term impact on the development of type 2 diabetes (T2D) mellitus in humans remains elusive. Thus, this study aimed to assess the cumulative incidence and association between thyroid disorders and T2D development.

**Methods:**

We conducted a retrospective cohort study using data from the Disease Analyzer database (IQVIA^™^, USA) from 2005 to 2022. The study included 158,674 patients with thyroid disorders and an equal number of matched patients without thyroid disorders. Propensity score matching was performed to balance age, sex and codiagnoses between the cohorts. Kaplan–Meier curves and Cox regression models were used to assess the cumulative incidence and hazard ratios (HRs) for new-onset T2D.

**Results:**

After a 10-year follow-up period, the cumulative incidence of T2D was higher in patients with thyroid disorders compared to the non-thyroid disorder cohort (*P* < 0.001). The HRs for T2D were 1.34 (95% CI: 1.28–1.39) for hypothyroidism and 1.30 (95% CI: 1.21–1.39) for hyperthyroidism. The strongest associations were observed in younger age groups for both hypothyroidism and hyperthyroidism.

**Conclusion:**

Thyroid disorders, including hypothyroidism and hyperthyroidism, are associated with an increased incidence of new-onset T2D. These findings suggest the need for proactive screening and management of glucose metabolism in patients with thyroid dysfunctions, particularly in younger individuals, independent of metabolic risk factors.

## Introduction

Thyroid dysfunction and diabetes mellitus are the two most common endocrine disorders, and they frequently co-occur in patients (1). While an association between autoimmune thyroid disease and type 1 diabetes mellitus has been well established (2), evidence also suggests a strong link between thyroid disorders and type 2 diabetes (T2D) mellitus. Population studies have shown a 10–22% higher prevalence of T2D in individuals with thyroid disorders (3). The Third National Health and Nutrition Examination Survey (NHANES III), a large cross-sectional survey of 17,353 participants in the USA, found hypothyroidism and hyperthyroidism in 4.6 and 1.3% of participants, respectively, with an increased incidence of thyroid dysfunction in people with diabetes (4). Similarly, large population-based longitudinal studies, mainly from iodine-rich Asia, have reported an increased prevalence of T2D in individuals with both hypo- and hyperthyroidism (5, 6, 7). A recent 5-year follow-up study from Germany and Denmark found that elevated thyroid hormone (e.g., hyperthyroidism) levels are associated with an increased incidence of T2D (8).

Thyroid hormones, free thyroxine (fT4) and triiodothyronine (fT3), are essential regulators of metabolism, influencing energy expenditure, lipid and glucose metabolism and insulin secretion (9, 10). They have also been found to support pancreatic β-cell viability and promote their proliferation (11).

While hyperthyroidism may initially improve insulin sensitivity, prolonged hyperthyroid states have been shown to lead to pancreatic β-cell exhaustion with reduced insulin secretion and impaired glucose tolerance (12). In addition, excess plasma thyroid hormone levels increase liver gluconeogenesis and peripheral insulin resistance, which in combination may eventually progress to T2D (13, 14). To date, the hyperthyroidism-induced metabolic stress is discussed to drive the pathogenesis of T2D over time, especially in individuals with preexisting metabolic vulnerabilities, such as insulin resistance or prediabetes (15). Although the negative impact of thyrotoxicosis on glucose metabolism has been known for decades, its exact prevalence and incidence remain a subject of debate, especially in iodine-deficient countries (16, 17).

Hypothyroidism, characterized by low levels of circulating thyroid hormones, has also been demonstrated to lead to decreased peripheral insulin sensitivity and glucose intolerance (18). In addition, hypothyroidism is associated with other metabolic disorders, such as dyslipidemia, hypertension and obesity, all of which may contribute over time to insulin resistance and the development of T2D (19). Notably, the treatment of hypothyroidism has been shown to improve insulin sensitivity, emphasizing the crucial role that thyroid hormones play in glucose metabolism (20). Although a shared pathogenesis involving impaired mitochondrial metabolism has been suggested, the underlying mechanisms and long-term contributing factors remain complex (21). A longitudinal study conducted in the Netherlands with a follow-up period of 7.9 years found that individuals with hypothyroidism had a significantly higher incidence of developing T2D than those with normal thyroid function (22). However, other studies have failed to find a significant association, highlighting the need for large-scale population-based investigations (23, 24).

Taken together, the relationship between thyroid dysfunction and T2D has been of increasing interest in recent years. Although numerous epidemiological studies have suggested an association between both elevated and decreased thyroid hormones and T2D, the results have been inconsistent for iodine-deficient countries, and most long-term data are from iodine-rich Asia (5, 6, 7, 24). Moreover, previous studies have typically focused on only one type of thyroid disorder (either hypothyroidism or hyperthyroidism) while often employing shorter observational periods. Therefore, the aim of this large retrospective cohort study is to investigate the cumulative incidence of T2D in individuals with hypothyroidism and hyperthyroidism over an extended 10-year follow-up period in iodine-deficient Germany. In addition, this study will also investigate the effects of different demographical and clinical factors on the association between thyroid disorders and T2D.

## Methods

### Database

This retrospective cohort study was based on data from the Disease Analyzer database (IQVIA^™^), which contains drug prescriptions, diagnoses and basic medical and demographic data obtained directly and in anonymous format from computer systems used in the practices of general practitioners and specialists (25). The database covers approximately 3000 outpatient clinic-based physicians in Germany. All clinical data have been coded using the German version of the International Classification of Diseases, 10th revision (ICD-10). It has previously been shown that the panel of practices included in the IQVIA^™^ database is representative of general and specialized practices in Germany (25). Finally, this database has been used in previous studies focusing on thyroid diseases (26) and diabetes mellitus (27, 28).

### Study population

**Figure 1:**
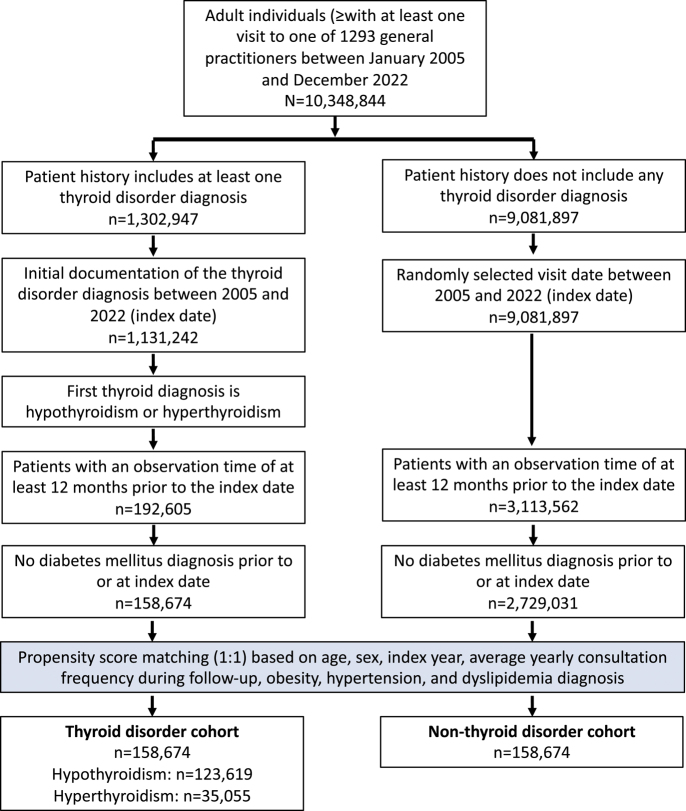
Flow chart of thyroid disorder and non-thyroid disorder cohort selection.

This study comprises two cohorts, a thyroid disorder cohort and a non-thyroid disorder cohort; a flow chart of the selection process is shown in Fig. 1. All patients included in both cohorts were aged ≥18 years from 1293 general practices in Germany between January 2005 and December 2022 (index date, Fig. 1). The thyroid disorder cohort included patients with a first documented diagnosis of hypothyroidism (ICD-10: E03) and hyperthyroidism (ICD-10: E05). Other thyroid disorders were excluded in the analyses, due to undefined origin (e.g., goiter), unclear thyroidal hormonal status (autoimmune thyroiditis, ICD-10: E06.3) or rare documentation.

The non-thyroid disorder cohort included patients without a documented thyroid disorder matched to thyroid disorder patients using nearest neighbor propensity score matching (1:1) based on age, sex, index year, average annual consultation frequency during follow-up and codiagnoses of obesity (ICD-10: E66), hypertension (ICD-10: I10) and dyslipidemia (ICD-10: E78) documented within 12 months before or on the randomly selected index date between January 2005 and December 2022 (Fig. 1).

For both groups, an inclusion criterion was an observation period of at least 12 months before the index date to access codiagnoses documented within 12 months before the index date. Patients with documentation of diabetes mellitus (ICD-10: E10-E14) before or on the index date were excluded in both groups.

**Figure 2:**
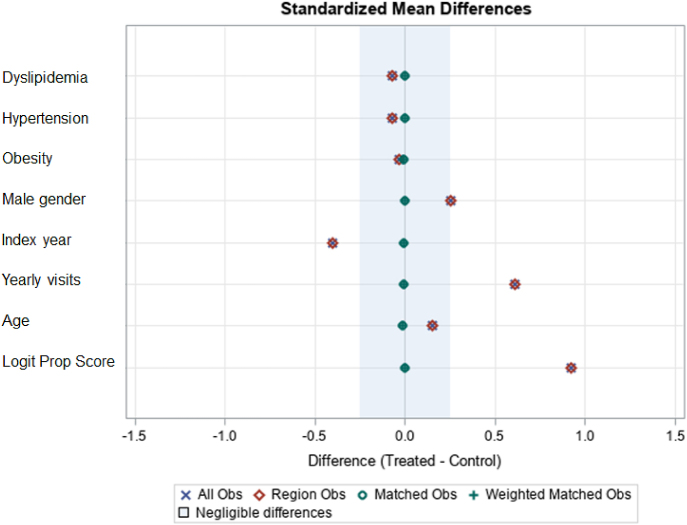
Propensity score matching balance of the thyroid disorder and non-thyroid disorder cohorts.

The standardized mean difference (SMD) was used to examine the balance of the covariate distribution between cohorts after matching. A SMD of less than 0.1 was allowed, indicating that adequate covariate balance was achieved (Fig. 2).

### Study outcomes

The outcomes of this study were the incidence of new-onset T2D (ICD-10: E11) in individuals with and without thyroid disorders (ICD-10: E03, E05), without previously known diabetes mellitus, over a 10-year observational time period after the index date.

### Statistical analyses

Descriptive statistics were reported as means (standard deviation) or numbers (percentage). The 10-year cumulative incidence of T2D was analyzed with Kaplan–Meier curves. Univariable Cox regression analysis was conducted to assess the association between each thyroid gland disorder and new-onset of T2D. These models were conducted separately for five age groups, sex and obesity. Results of the Cox regression model have been displayed as hazard ratios (HRs) and 95% confidence intervals (CIs). A *P*-value of <0.01 was considered statistically significant due to multiple comparisons to account for the potential inflation of type I error (29, 30). Analyses were conducted using SAS version 9.4 (SAS Institute, USA).

## Results

### Basic characteristics of patients with and without thyroid disorders

This study included a total of 158,674 individuals in the thyroid disorder cohort and the same number (158,674) in the non-thyroid disorder cohort. In the thyroid disorder cohort, there were 123,619 individuals diagnosed with hypothyroidism and 35,055 with hyperthyroidism (Fig. 1). In Fig. 2, the results of the propensity score matching are displayed, which achieved a SMD of less than 0.01, indicating well-balanced covariates between the thyroid and non-thyroid disorder cohorts.

The mean baseline characteristics of the study population are given in Table 1. The median follow-up times (interquartile range (IQR) for the cohorts were as follows: hypothyroidism, 1494 days (IQR: 2245 days); hyperthyroidism, 1535 days (IQR: 2399 days); and non-thyroid disease, 1290 days (IQR: 1939 days). The mean age was 49 years in patients with hypothyroidism and 56 years in patients with hyperthyroidism. The number of physician visits per year was 6 to 7 among the groups. The majority of patients were female, accounting for 73% of those with hypothyroidism and 68% of those with hyperthyroidism. The prevalence of obesity (BMI >30 kg/m^2^) was similar between groups, ranging from 6.9 to 9.5%, while dyslipidemia affected 21% patients with hypothyroidism and 24% with hyperthyroidism. The prevalence of hypertension was lower among patients with hypothyroidism (29.9%) than among patients with hyperthyroidism (39.5%) (Table 1).

**Table 1:** Baseline characteristics of the study population categorized as hypothyroidism and hyperthyroidism with their matched control pair after 1:1 propensity score matching.

Variable	Patients with hypothyroidism (*n*, %)	Matched pairs without hypothyroidism (*n*, %)	Patients with hyperthyroidism (*n*, %)	Matched pairs without hyperthyroidism (*n*, %)
	*n* = 123,619	*n* = 123,619	*n* = 35,055	*n* = 35,055
Age [mean, SD]	48.8 [18.4]	49.2 [18.5]	56.0 [18.2]	56.0 [18.3]
Age 18–40	45,701 [37.0]	44,763 [36.2]	8041 [22.9]	8.073 [23.0]
Age 41–50	21,561 [17.4]	21,432 [17.3]	5314 [15.2]	5315 [15.2]
Age 51–60	21,947 [17.7]	22,118 [17.9]	6774 [19.3]	6761 [19.3]
Age 61–70	16,136 [13.1]	16,357 [13.2]	5903 [16.8]	5911 [16.9]
Age >70	18,274 [14.8]	18.949 [15.3]	9023 [25.7]	8995 [25.6]
Number of physician visits/year	6.2 [4.2]	6.2 [4.2]	7.0 [4.4]	7.0 [4.4]
Female	90,525 (73.2)	90,919 (73.5)	23,738 (67.7)	23,845 (68.0)
Male	33,094 (26.8)	32,700 (26.5)	11,317 (32.3)	11,210 (32.0)
Obesity	11,747 (9.5)	10,526 (8.5)	2415 (6.9)	2329 (6.6)
Dyslipidemia	25,994 (21.0)	25,585 (20.7)	8451 (24.1)	8370 (23.9)
Hypertension	36,989 (29.9)	37,037 (30.0)	13,865 (39.5)	13,770 (39.3)

Proportions of study population are given in *n*, %, unless otherwise indicated. SD, standard deviation.

### Cumulative incidence of T2D in patients with and without thyroid disorders

**Figure 3:**
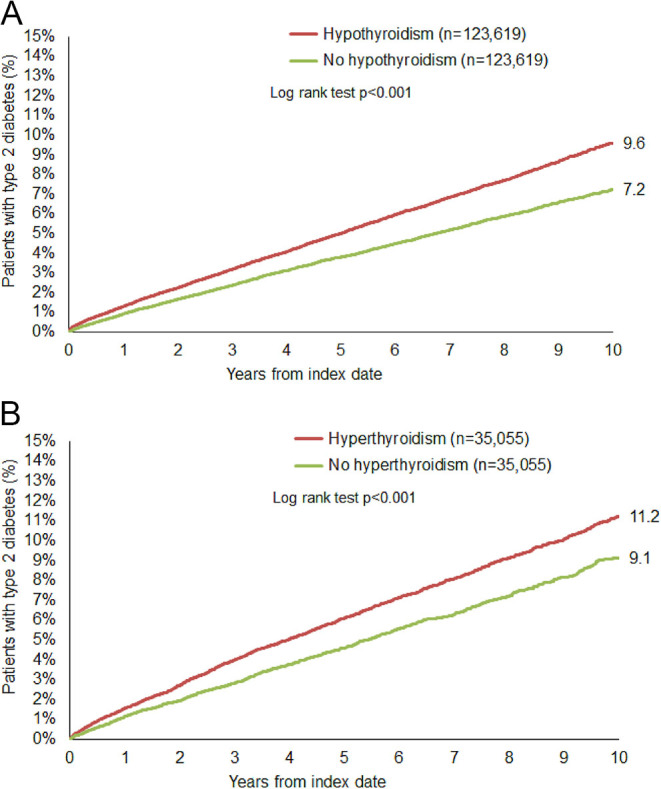
Cumulative incidence of new-onset type 2 diabetes mellitus in the thyroid disorder (red) and non-thyroid disorder (green) cohorts over a 10-year time period.

Over a 10-year follow-up period, the cumulative incidence of T2D was significantly higher in the thyroid disorder cohort than in the non-thyroid disorder cohort (Fig. 3). With hypothyroidism, T2D developed over 10 years in 9.6% of patients compared to 7.2% in the paired no hypothyroidism group (*P* < 0.001). In the hyperthyroidism group, 11.2% of patients presented new-onset T2D compared to 9.1% in the paired no hyperthyroidism group (*P* < 0.001) (Fig. 3).

### Association between thyroid disorders and subsequent T2D

In the regression analysis, an association was observed between both forms of thyroid disorders (hypothyroidism and hyperthyroidism) and new-onset T2D in the overall population over a 10-year time period (all *P* < 0.001). The HR for T2D was as follows: hypothyroidism (HR: 1.34; 95% CI: 1.28–1.39) and hyperthyroidism (HR: 1.30; 95% CI: 1.21–1.39) (Table 2).

**Table 2:** Association between thyroid disorders (hypothyroidism; hyperthyroidism) and new-onset type 2 diabetes and predefined variables in patients followed up for 10 years in general practices in Germany (univariable Cox regression models).

Patient subgroup	Hypothyroidism		Hyperthyroidism	
	HR (95% CI)	*P* value	HR (95% CI)	*P* value
Total	1.34 (1.28–1.39)	**<0.001**	1.30 (1.21–1.39)	**<0.001**
Age 18–40	1.67 (1.47–1.89)	**<0.001**	1.59 (1.15–2.20)	**0.005**
Age 41–50	1.47 (1.33–1.63)	**<0.001**	1.44 (1.17–1.76)	**<0.001**
Age 51–60	1.28 (1.18–1.38)	**<0.001**	1.19 (1.04–1.36)	**0.013**
Age 61–70	1.32 (1.22–1.44)	**<0.001**	1.19 (1.04–1.35)	1.011
Age >70	1.35 (1.24–1.47)	**<0.001**	1.45 (1.28–1.64)	**<0.001**
Female	1.38 (1.31–1.45)	**<0.001**	1.35 (1.24–1.48)	**<0.001**
Male	1.28 (1.20–1.37)	**<0.001**	1.24 (1.11–1.38)	**<0.001**
Individuals with obesity	1.24 (1.13–1.35)	**<0.001**	1.23 (1.03–1.46)	**0.025**
Individuals without obesity	1.34 (1.28–1.40)	**<0.001**	1.30 (1.21–1.40)	**<0.001**

HR, hazard ratio. Bold indicates statistical significance.

The strongest association between hypothyroidism and T2D was observed in the 18- to 40-year age group (HR: 1.67; 95% CI: 1.47–1.89). Similarly, the strongest association between hyperthyroidism and T2D was also found in the 18- to 40-year age group (HR: 1.59; 95% CI: 1.15–2.20). However, hyperthyroidism was not associated with T2D in the 61- to 70-year age group (HR: 1.19; 95% CI: 1.04–1.35).

The prevalence of both thyroid disorders with T2D was observed in women (hypothyroidism HR: 1.38; 95% CI: 1.31–1.45; hyperthyroidism HR: 1.25; 95% CI: 1.24–1.48) and men (hypothyroidism HR: 1.28; 95% CI: 1.20–1.37; hyperthyroidism HR: 1.24; 95% CI: 1.11–1.38), with no relevant differences between the sexes. Interestingly, in individuals with obesity, hypothyroidism (HR: 1.24; 95% CI: 1.13–1.35) and hyperthyroidism (HR: 1.23; 95% CI: 1.03–1.46) were associated with T2D. Similarly, in people without obesity (BMI <30 kg/m^2^), the different thyroid conditions (hypothyroidism HR: 1.34; 95% CI: 1.28–1.40; hyperthyroidism HR: 1.30; 95% CI: 1.21–1.40) were both associated with T2D over ten years.

## Discussion

This large retrospective cohort study evaluated the association between thyroid gland disorders and T2D over a 10-year time period in Germany. Our results revealed that individuals with thyroid disorders, including hypothyroidism and hyperthyroidism, had a higher incidence of T2D than those without thyroid disorders. The cumulative incidence of T2D was increased in both thyroid disorders over the 10-year follow-up period, with the highest incidence observed in younger people with both hypothyroidism and hyperthyroidism. Men and women were similarly affected, and the association between thyroid disorders and T2D was present in both obese and non-obese individuals. Our findings reveal that thyroid disorders are associated with T2D development and highlight the need for proactive age-dependent metabolic monitoring in patients with thyroid disorders.

Our study shows that 9.6 and 11.2% of patients with hypothyroidism and hyperthyroidism, respectively, developed T2D over a 10-year period, compared with 7.2 and 9.1% in their matched controls without thyroid disorders. These findings are surprisingly well in line with a previous non-iodine-deficient population, which reported a 9.2% higher prevalence of T2D in individuals with thyroid dysfunction in the Tabari population (7). In addition, a meta-analysis found a 17% higher rate of T2D in patients with hypothyroidism and hyperthyroidism, with a J-shaped relationship between thyroid-stimulating hormone (TSH) levels and T2D prevalence (31). Notably, another large-scale meta-analysis of thyroid hormone levels, cardiovascular diseases and mortality also identified a J-shaped association between fT4 and these outcomes, which underlines the clinical importance (32). In a Dutch cohort study, the risk of T2D was 1.16 times higher with elevated TSH levels and, for every doubling of TSH levels, the T2D prevalence increased by 1.09 times (22). Furthermore, a 7-year follow-up study in euthyroid individuals revealed that fluctuations in TSH and thyroid hormones, even within the normal reference range, were an additional risk factor for the onset of T2D (5).

A novel aspect of our findings is the age-specific association between different types of thyroid dysfunction and T2D. Younger individuals (18–40 years) with hypothyroidism and hyperthyroidism have a higher incidence of T2D. This age group seems particularly vulnerable to the metabolic changes induced by thyroid dysfunction, such as insulin resistance and hyperglycemia. A previous study similarly identified a higher prevalence of T2D in younger females, particularly within the first 0.5–1 year after the initial diagnosis of thyroid dysfunction (6). In line with this, a cross-sectional, population-based study postulated to introduce age-specific TSH reference ranges, given that TSH levels tend to increase with age, to avoid overdiagnosis of subclinical hypothyroidism in the elderly (33). These findings underscore the critical role of age in understanding the relationship between thyroid dysfunction and T2D incidence. Age-specific screening and preventive strategies may be warranted to prevent T2D in these high-risk groups.

Interestingly, the association between thyroid dysfunction and T2D was observed in both men and women, with no differences between the sexes. Thus, thyroid dysfunction appears to potentiate T2D development regardless of sex. However, it has been well established that the prevalence of thyroid disorders, including hyperthyroidism and hypothyroidism, is higher in women than in men (4). In iodine-deficient populations, the prevalence of these disorders ranges from 0.5 to 4%, yet women are approximately 7–8 times more likely to be diagnosed with a thyroid dysfunction than men (34). This disparity is primarily due to strong hormonal fluctuations, including puberty, pregnancy and menopause, which can impact thyroid function (34). Estrogen enhances immune responses, thereby increasing the likelihood of conditions such as Hashimoto’s thyroiditis and Graves’ disease, which can lead to thyroid disorders (34, 35). Women might also be more frequently screened for thyroid conditions during these stages of life (34). In our study population, 68–83% of participants with thyroid dysfunction were female, while only 17–32% were male, reflecting the sex disparity in thyroid disease prevalence. Consistent with our findings, a cross-sectional study of people with thyroid disorder showed a prevalence of T2D of 16.2% in men and 7.7% in women, also with no sex differences (37). Moreover, thyroid hormones have been shown to influence T2D incidence, with lower fT3 and fT3/fT4 ratio and higher fT4 levels being associated with increased T2D prevalence in both sexes. However, TSH has also been shown to be inversely related to T2D in males, but not in females (36).

Obesity is a well-known risk factor for both T2D and thyroid disorders, especially hypothyroidism (26). However, our findings show that those individuals with both hypothyroidism and hyperthyroidism have increased incidence of T2D independent of the presence of obesity. In line with this, a previous 10-year follow-up study found fT4 to be a significant predictor of the metabolic syndrome, but only for non-obese people (37). However, obesity was reported in only 6.6–9.5% of this study population. General population statistics report 19% obesity in Germany; thus, the low prevalence might influence our results (38). This discrepancy may result from the study design or its retrospective character. However, thyroid disorders may independently of obesity contribute to metabolic disturbances, reinforcing the need for proactive T2D screening regardless of the body weight (12).

One key strength of this study is its use of a large, population-representative data sample from Germany, which enhances the reliability and generalizability of the findings. In contrast, previous epidemiological studies have typically involved 100 times smaller sample sizes (8, 24). However, several limitations of this cohort study must be noted. First, the retrospective design may introduce potential biases connected to observational research. Although we used propensity score matching to balance key variables between the thyroid disorder and non-thyroid disorder cohorts, unmeasured confounders, such as family history of diabetes, dietary habits, physical activity, genetic predisposition and ethnicity, could not be taken into account in this study. The German Disease Analyzer database also does not capture detailed laboratory values or treatment status for all study patients. In addition, the observational design of this study is not intended or equipped to directly investigate biological mechanisms between thyroid disorders and T2D. Then, coded data of clinical diagnoses may lead to underestimation of certain conditions (i.e., obesity). Future studies are needed to support our findings and investigate the reciprocal relationship between thyroid disease and T2D in greater depth.

In conclusion, our 10-year cohort study provides novel insights into the complex bidirectional relationship between thyroid dysfunction and the incidence of T2D in Germany. We demonstrate that both hypothyroidism and hyperthyroidism were associated with an elevated incidence of developing T2D, particularly in younger individuals. These findings underscore the importance of metabolic monitoring, age-specific screening and preventive strategies in patients with thyroid disorders to diminish the incidence of T2D.

## Declaration of interest

The authors declare no conflict of interest regarding the actual study.

## Funding

This work did not receive any specific grant from any funding agency in the public, commercial or not-for-profit sector.

## Data availability

The datasets used and analyzed during the current study are available from the corresponding author on reasonable request.

## Institutional review board statement

Ethical review and approval were waived for this study, because the database used for analysis contains anonymized electronic patient records. Patient data were analyzed in aggregated form without individual data being available.

## Informed consent statement

Individual consent forms were not required or obtained, in accordance with national and European legislations.
